# Peer-review ownership in the AI era

**DOI:** 10.1038/s44319-026-00706-7

**Published:** 2026-02-03

**Authors:** Christos A Ouzounis

**Affiliations:** BCCB Group, AIIA Lab, School of Informatics, Aristotle University of Thessalonica, Thessalonica, Greece

**Keywords:** Science Policy & Publishing

## Abstract

The peer review process is fundamental to scientific validation, yet ownership and permitted uses of the resulting content remain insufficiently defined. To ensure the future integrity and impartiality of manuscript assessment, journals must consider updating community agreements that define the reuse rights of reviewers and establish mechanisms for consent or compensation.

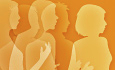

The peer review process is essential to scientific publishing (Rowland, 2002). Nonetheless, the ownership and permitted uses of reviews, whether anonymous or published alongside a manuscript, remain slightly ambiguous. As the drive for transparency in peer review grows and the nature of scientific publication evolves, the question of who controls the reuse of review texts and associated metadata becomes vital. While academic publishers and scientific journals are experimenting with a range of peer-review models, further debate is needed on the rights associated with review texts, particularly once they are published.

Historically, peer review has operated under conditions of confidentiality and anonymity. In order to maintain the requirement of objectivity, reviewers assess articles without disclosing their identity to the author(s). Since only the authors and editors normally have access to their reviews, ownership has not been an issue. The primary function of confidential reviews is to guide editorial decisions and to improve the manuscript, and these texts are in principle discarded by editors after publication. But as publishing practices have advanced (Horbach and Halffman, 2018), so should our understanding of peer-review ownership, especially beyond the publication process.

In the confidential peer-review mode, reviewers may generally retain ownership of their reports. They share their knowledge on a voluntary basis and typically anticipate that this knowledge stays under their control. That includes options for reusing it (Drozdz and Ladomery, 2024). Yet this assumption is seldom stated, leaving reviewers exposed to uncertainty and potential legal predicaments. Consider a case where a reviewer wants to recycle parts of a report in their own work—perhaps to refine a key methodological critique for inclusion in a review article, a textbook chapter, or even a conference presentation. Reviewers may rightly wonder if they need permission to proceed.

Publishers may handle reviews not just as reports on which to make decisions, but also as valuable data assets. This new trend toward proprietary control by publishers and the old expectation of ownership by reviewers is now being challenged: the rise of AI agents such as qed offering automated reviews signals that the reuse of content by third parties is no longer a theoretical option. While the immediate financial value of individual reports for AI training might be modest—with the exception of large publishing companies where cost-cutting has evident effects—the primary argument remains one of transparency and the “right thing to do” for the integrity of research assessment. Furthermore, the emergence of reviewer credit initiatives reflects a growing recognition that this labor must be quantified somehow, yet such systems can only succeed if the underlying ownership is clearly established.

Open peer review adds even more complications as some journals release review texts next to published articles, sometimes revealing the identity of reviewers (Tennant and Ross-Hellauer, 2020). Here, a distinction must be made between Open Review, which mandates reviewer identification and Transparent Review, which was pioneered by EMBO Press, where the publication of reports and names is handled on a voluntary basis. This difference is critical, as it allows for public accountability while preserving the reviewer’s agency over their identity. Publishers usually ask for narrow, limited licenses to use reviews as part of the editorial process; they rarely claim full ownership. Still, publishing a review —often with its own DOI—raises questions about the review’s legal status as a standalone scholarly item as it could belong to the reviewer, the publisher or both, or enters the public domain under a CC license with unclear rights about who is allowed to do what.

Published, accessible reviews with a DOI count as full citable items on their own. Researchers can indeed list “open reviews” among their academic profiles (Tennant, 2018). When a review also includes the reviewer’s name, it is in fact similar to a standard article with the author as the copyright holder. On the flip side, anonymous reviews reverse that pattern: without credit attached, reviewers, intentionally, cannot claim copyrights even though they retain intellectual property rights.

There are other issues to consider (Waltman et al, 2023). Journals may argue that they have the right to publish reviews for transparency, but this does not imply ownership of the underlying data. Proprietary submission systems allow publishers to create internal high-value datasets linking manuscripts, reviews, author responses, editorial decisions, review time, and so on. With the rise of AI, not only the text but also the associated metadata become a valuable asset. Such metadata are not copyrightable, which further weakens claims of exclusive rights over them. Reviewer agreements rarely, if ever, contain an explicit clause preventing publishers from using these composite datasets to train their machine-learning models. As a result, the current legal ambiguities allow publishers to potentially monetize the composite dataset of intellectual critique and behavioral/editorial metadata by creating profitable AI-driven systems, effectively bypassing the copyright holder, without providing explicit compensation or securing consent on a case-by-case basis.

Reviewers should therefore be made aware of the conditions under which their reviews will be utilized, particularly if they are made public (PLoS Biology, 2019). On the other side, reviewers sometimes decide to contribute freely to the advancement of science, regardless of attribution, recognizing that their reviews are used for the sake of scholarly validation. Even here, transparency about rights and reuse is essential, for reasons outlined above.

The ownership of peer reviews remains poorly defined, particularly as boundaries between anonymous and publicly accessible evaluations blur. Journals should set clearer guidelines, so reviewers actually understand their rights and obligations, especially when reviews become part of a data product. Agreements should be updated to specify how the content and metadata are used for AI training, offering clear opt-out or compensation mechanisms. As open peer-review continues to expand, resolving these emerging issues will be critical to ensure the integrity and impartiality of the peer-review process (Berenbaum, 2023). The traditional model has long provided stability and reliability (Alberts et al, 2008), with or without AI. However, its persistence may be a historical accident whose era may now be ending. As someone who has reviewed or edited hundreds of scientific papers, with a strong preference for anonymous reviews, I keep wondering how the new generation of scientists will adapt to this complex, evolving landscape of manuscript assessment.

## Editor’s note

*EMBO Press* mandates that referees name co-referees, use transparent review, and encourage refereed preprints.

## Supplementary information

Peer Review File
