# Supplementary material for: Peer-review ownership in the AI era
Source: EMBO Rep. 2026 Feb 3;27(4):829–30. doi: 10.1038/s44319-026-00706-7 (PMC12936054; doi:10.1038/s44319-026-00706-7)
Supplement: Supplementary file 1 — Peer Review File [file 44319_2026_706_MOESM1_ESM.pdf]

EMBO reports

Peer Review Ownership in the AI Era

Christos Ouzounis

Corresponding author(s): Christos Ouzounis (cao@csd.auth.gr)

Review Timeline:

Submission Date:	23rd Jan 26
Accepted:	26th Jan 26

Editor: Holger Breithaupt

Transaction Report: This article type is not peer reviewed.
